# Multiplex coherent anti-Stokes Raman scattering highlights state of chromatin condensation in CH region

**DOI:** 10.1038/s41598-019-50453-0

**Published:** 2019-09-25

**Authors:** Tiffany Guerenne-Del Ben, Zakaniaina Rajaofara, Vincent Couderc, Vincent Sol, Hideaki Kano, Philippe Leproux, Jean-Michel Petit

**Affiliations:** 1 PEIRENE, EA 7500, University of Limoges, 123 avenue Albert Thomas, 87060 Limoges, France; 2 XLIM, UMR 7252, University of Limoges, 123 avenue Albert Thomas, 87060 Limoges, France; 3 Department of Applied Physics, Graduate School of Pure and Applied Sciences, University of Tsukuba, 1-1-1 Tennodai, Tsukuba, Ibaraki 305-8573 Japan; 4 Institute of Applied Physics, University of Tsukuba, 1-1-1 Tennodai, Tsukuba, Ibaraki 305-8573 Japan; 5 LEUKOS, 37 rue Henri Giffard, 87280 Limoges, France

**Keywords:** Chromosome condensation, Mitosis, Cellular imaging

## Abstract

Coherent Raman microscopy has become a powerful tool in label-free, non-destructive and fast cell imaging. Here we apply high spectral resolution multiplex coherent anti-Stokes Raman scattering (MCARS) microspectroscopy in the high wavenumber region to the study of the cell cycle. We show that heterochromatin - the condensed state of chromatin - can be visualised by means of the vibrational signature of proteins taking part in its condensation. Thus, we are able to identify chromosomes and their movement during mitosis, as well as structures like nucleoli and nuclear border in interphase. Furthermore, the specific organization of the endoplasmic reticulum during mitosis is highlighted. Finally, we stress that MCARS can reveal the biochemical impact of the fixative method at the cellular level. Beyond the study of the cell cycle, this work introduces a label-free imaging approach that enables the visualization of cellular processes where chromatin undergoes rearrangements.

## Introduction

In the last two decades, Raman microspectroscopy has become a powerful tool in label-free cell imaging. It could for example reveal cellular components in neutrophils, such as a multilobed nucleus, lipid droplets, nucleoli and mitochondria^1^. It made also possible to identify the endoplasmic reticulum, the Golgi apparatus and intracellular vesicles in HeLa cells^2^. Basically, such identifications are realised by means of the vibrational signature of lipids and proteins. However the sole lipid signature could be used to investigate cell lines such as cancer cells^3,4^ and to differentiate proliferating cells from senescent ones through modifications of the nuclear envelope^5^. Recently Raman microspectroscopy was applied to evaluate the impact of an anti-cancer treatment^6^.

On the other hand, Raman imaging has some drawbacks that limit its dissemination. Namely, the vibrational signal overlaps with autofluorescence, which is inherent in biological samples. This results in a reduction of the signal-to-noise ratio. In connection with this aspect and due to the spontaneous nature of Raman scattering, the analysis time remains long and is generally not consistent with the observation of living cells. To avoid this limitation, the use of coherent anti-Stokes Raman scattering (CARS) microscopy is particularly appropriate. Since the demonstration of its high sensitivity and high spatial resolution in 1999^7^, CARS microscopy has been extensively employed to image prokaryotes, mammal and plant cells, human tissues, etc. Commonly, CARS technology is used to analyse lipid content in cells through the strong vibrational signature of methylene (CH_2_) groups at 2850 cm^−1 ^^7,8^. Using this signature, authors could monitor the differentiation of fetal skeletal stem cells in adipogenic or chondrogenic cells^9^, the uptake of lipids in monocytes^10^ or in fibroblasts^11^. Others characterized murine stem cells during their differentiation into adipocytes^12^. A link between lipid-rich tumour cells and their aggressive comportment was established by CARS analysis^13^. More specifically, it was possible to identify circulating tumour cells using the specific signature of lipids^14^.

In a CARS experiment, the sample is illuminated with two laser waves simultaneously, namely the pump (ω_p_ frequency) and Stokes (ω_S_ frequency) waves. When the difference ω_p_ − ω_S_ is equal to the vibration frequency of the molecular bond of interest, the resonant CARS signal is generated at frequency 2ω_p_ − ω_S_. CARS being a coherent process, the signal intensity can be strongly enhanced compared to spontaneous Raman scattering, allowing to reduce the analysis time^9,15^.

In order to generate the resonant CARS signal over the full range of Raman shifts (roughly 500–3500 cm^−1^, including both the fingerprint, CH and OH stretching regions) simultaneously, it is necessary to use a Stokes wave having a very broad spectrum at wavelengths longer than that of the pump. Such radiation can be obtained by generating a supercontinuum in a photonic crystal fibre (PCF)^16^. By this way, and by ensuring the synchronicity between the pump and Stokes pulse trains, it is possible to implement so-called “multiplex CARS” (MCARS)^17,18^ or “broadband CARS” (BCARS)^19,20^ microspectroscopy. These configurations can then provide rich vibrational information from biological samples.

To date CARS microscopy/microspectroscopy has been massively applied to the label-free imaging of fixed or living cells, highlighting its ability to discern intracellular constituents such as nucleus, nucleoli, mitochondria, etc. However, these studies generally do not take into account the fact that proliferating cells *in vitro* constitute a heterogeneous cell population, mainly due to cell cycle^21^, during which all cell types change their DNA, RNA, lipid and protein contents. During cell cycle, the cells pass through four distinct phases: G_1_, S, G_2_ and M. G_1_ and G_2_ phases are characterized by protein and RNA syntheses in order to prepare cells to S phase (DNA synthesis) then M phase (mitosis)^22^ respectively. Moreover, the location and organisation of chromatin differs according to M sub-phases: (1) during prophase the chromosomes become observable due to a higher level of chromatin condensation, and the nuclear envelope dissociates; (2) the prometaphase is characterized by the chromosome capture by microtubules, and by the organization of mitotic spindle; (3) in metaphase chromosomes are aligned at the metaphase plate; (4) during anaphase, the sister chromatids of each chromosome separate; (5) telophase corresponds to the end of mitosis with chromatin decondensation and nuclear envelope restructuration; cytokinesis marks the end of cell division.

Since the work by Matthäus *et al*. in 2006 based on spontaneous Raman scattering^23^, very few studies of cell cycle have been realised by means of coherent Raman microscopy. In 2010, CARS imaging of proteins (2928 cm^−1^) and lipids (2840 cm^−1^) in live mitotic and interphase cells was done, but no correlation was observed between the DNA compaction level and the proteins’ signal^24^. Recently, cell division (prometaphase, anaphase, telophase) was investigated by using CARS in the CH stretching region, as well, and by applying a cumbersome data analysis method rendering images of water, proteins, DNA/proteins and lipids^25^. The reproducibility of the study was considered by merely analysing a second prometaphase cell. Moreover, this study is somewhat reminiscent of a previous work where linear decomposition of multicolour stimulated Raman scattering (SRS) data was used to image DNA in live mitotic and interphase cells^26^. Finally, MCARS with 600–3200 cm^−1^ coverage could be used to achieve molecular fingerprinting of living cells at different phases of the cell cycle^27^. Such spectral coverage includes many vibrational bands in the fingerprint and CH stretching regions with selectivity to DNA/RNA, proteins and lipids.

In this work, we apply MCARS microspectroscopy in the CH stretching vibrational region (2500–3200 cm^−1^) with high spectral resolution (<1 cm^−1^) to the study of cell cycle, including a high number of fixed and living cells in interphase and all sub-phases of mitosis. We show that, throughout the cell cycle, heterochromatin - the condensed state of chromatin - can be visualised in the CH_3_ stretching band by means of the vibrational signature of proteins that take part in its condensation. This allows, in particular, localising chromosomes in mitotic cells. Significant spatial discrimination is demonstrated between the CH_3_ signal, predominantly localised in the nucleus, and the CH_2_ signal that highlights a greater quantity of lipids in the cytoplasm. Moreover, we look into the impact of cell fixation using paraformaldehyde (PFA) on the vibrational signature, with living cells as a reference. This work shows that high spectral resolution MCARS in the CH region is a user-friendly, label-free method enabling the observation of DNA/protein/lipid content with high contrast and short time, suitable for the analysis of living cells.

## Results

### Verification of cell cycle distribution and morphology

**Figure 1:**
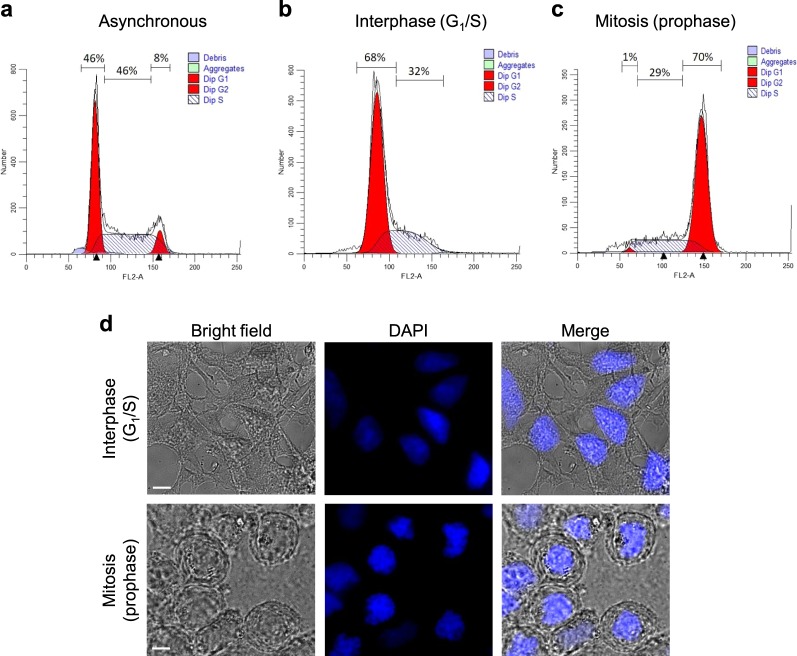
Analysis of HEK293 cell cultures by means of flow cytometry and conventional optical microscopy. (**a–c**) Flow cytometry histograms of asynchronous, interphase (G_1_/S) and mitosis (prophase) populations. Percentages correspond to cells in each cycle phase. (**d)** Bright-field and fluorescence microscopy images of the same interphase and mitosis populations. Scale bar, 10 µm.

Figure 1 displays the results of flow cytometry analysis for different HEK293 cell cultures. For asynchronous culture, 46% of cells were in G_1_ phase, 46% in S phase and only 8% in G_2_/M phases (Fig. 1a). After double thymidine block, a large number of cells (68%) was in G_1_ phase, versus 32% in S phase (Fig. 1b). As a matter of fact, the thymidine treatment is unable to block all cells at the G_1_/S boundary, but it can be concluded that all cells were in interphase. After thymidine and nocodazole treatment, 70% of cells were arrested in M phase (Fig. 1c).

Furthermore, we analysed cell cultures after DAPI staining by means of bright-field and fluorescence microscopies, which allow visualising the morphology of cells and nuclei, respectively (Fig. 1d). For interphase (G_1_/S) cells, a typical spread morphology is observed; the nucleus is well delimitated, and its staining is heterogeneous. In contrast, mitotic cells exhibit a preferably round shape^28^; the staining reveals the presence of hypercondensed chromatin (heterochromatin), which contributes to chromosome formation. Moreover, the overall higher fluorescence intensity is characteristic of an abundant binding of DAPI to more condensed DNA. It is noticeable that cells arrested in M phase are prophase cells.

### Analysis of fixed cells in interphase (G_1_/S) and mitosis (prophase)

**Figure 2:**
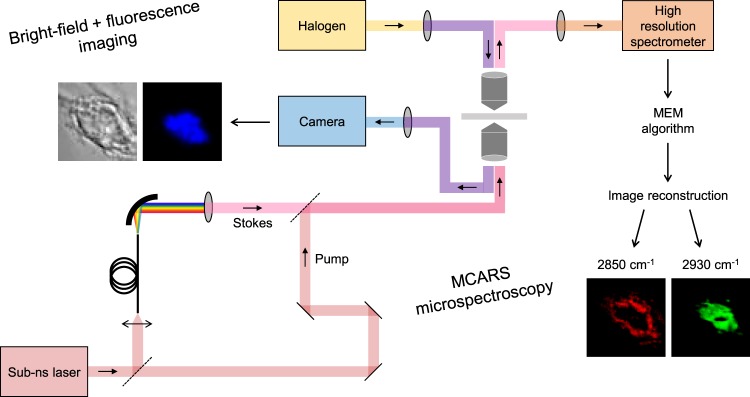
Experimental and numerical methodology for the analysis of cell cultures. First, cells of interest were selected using bright-field and/or fluorescence imaging. Then selected cells were mapped by means of high spectral resolution (<1 cm^−1^) MCARS microspectroscopy in the 2500–3200 cm^−1^ range (see Methods for details of the MCARS system). Finally, the vibrational information was extracted by using MEM algorithm, and cell images were reconstructed at 2850 cm^−1^ (CH_2_ symmetric stretching) and 2930 cm^−1^ (CH_3_ symmetric stretching).

Figure 2 illustrates the experimental and numerical methodology used for analysing HEK293 cell cultures. First, cells of interest were selected using bright-field and/or fluorescence imaging, depending on whether or not they were stained with DAPI. Then selected cells were mapped by means of high spectral resolution (<1 cm^−1^) MCARS microspectroscopy in the 2500–3200 cm^−1^ range. Finally, the vibrational information was extracted by using the maximum entropy method (MEM)^29^, and cell images were reconstructed at 2850 cm^−1^ (CH_2_ symmetric stretching) and 2930 cm^−1^ (CH_3_ symmetric stretching).

**Figure 3:**
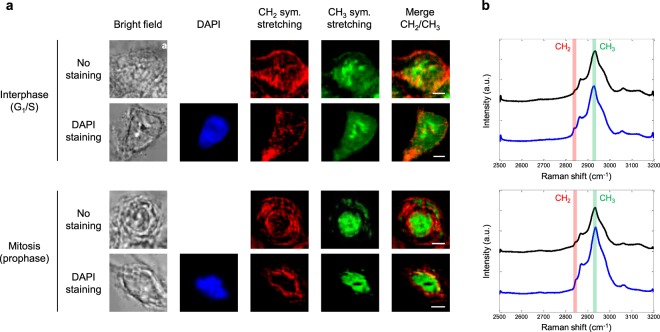
Analysis of interphase and mitotic, stained and unstained, fixed HEK293 cells. (**a**) Bright-field, fluorescence and MCARS (CH_2_, CH_3_, merge) images of selected cells. MCARS images were reconstructed at 2850 cm^−1^ (CH_2_ symmetric stretching) and 2930 cm^−1^ (CH_3_ symmetric stretching). Scale bar, 5 µm. (**b)** Standard deviation of the vibrationally resonant CARS signal between 2500 and 3200 cm^−1^, computed over the whole analysis window of each cell. Spectra are plotted vertically in the same order as corresponding cells. CH_2_ and CH_3_ channels are highlighted.

We investigated interphase and mitotic, stained and unstained, HEK293 cells. Figure 3a shows the images obtained in each case. On the whole, CH_2_ and CH_3_ vibrational signatures do not overlap and thus bring complementary information from the sample. The CH_2_ signal - highlighting lipids - is mainly localised in the cytoplasm, when the CH_3_ signal - primarily associated with proteins - appears predominantly in the nucleus. The difference in CH_2_ signal distribution between interphase and mitosis would indicate the specific organization of the endoplasmic reticulum (ER) during these phases and will be discussed subsequently. Regarding the CH_3_ signal, bright spots corresponding to nucleoli are observed inside the nucleus of interphase cells, while the signal extends more homogeneously to the nucleus in mitotic cells. These findings are similar for stained and unstained cells, since the Raman signature of DAPI labelling is expected in the fingerprint region^30,31^. To confirm this, we computed the standard deviation of the vibrationally resonant CARS signal between 2500 and 3200 cm^−1^ over the whole analysis window of each cell, as plotted in Fig. 3b. The standard deviation spectra have the same profile in the CH stretching range, and no additional band arises because of DAPI staining. Thus, stained and unstained cells can be indifferently studied by MCARS microspectroscopy in the 2500–3200 cm^−1^ range.

**Figure 4:**
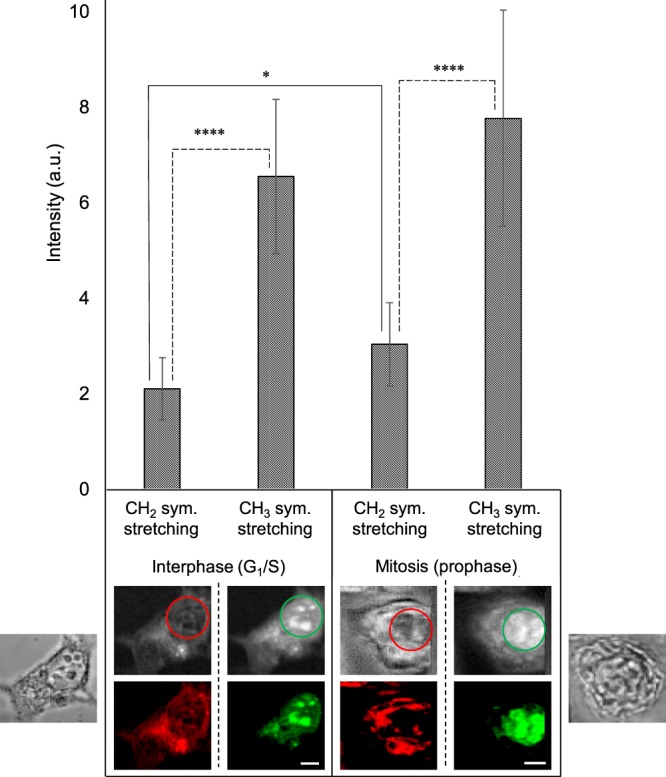
Quantification of the contrast between CH_2_ and CH_3_ signal intensities in the nucleus of fixed HEK293 cells. Lower panel represents bright-field and MCARS (CH_2_, CH_3_) images of one interphase (G_1_/S) cell and one mitotic (prophase) cell. For each cell, an ROI was drawn in the area of strong CH_3_ signature, then CH_2_ and CH_3_ signal intensities were integrated in this ROI. This method was applied to 10 cells in interphase (5 stained, 5 unstained, see Supplementary Fig. 1) and 10 cells in mitosis (4 stained, 6 unstained, see Supplementary Fig. 2), throughout three independent experiments. Scale bar, 5 µm. Upper panel displays the mean value of CH_2_ and CH_3_ integrated intensity for this set of interphase and mitotic cells.

Next, we quantified the contrast between CH_2_ and CH_3_ signal intensities in the nucleus for a set of 10 cells in interphase (5 stained, 5 unstained, see Supplementary Fig. 1) and 10 cells in mitosis (4 stained, 6 unstained, see Supplementary Fig. 2). For each cell, a region of interest (ROI) was drawn in the area of strong CH_3_ signature (Fig. 4, lower panel), then both CH_2_ and CH_3_ signal intensities were integrated in this ROI. Figure 4 (upper panel) displays the mean value of CH_2_ and CH_3_ integrated intensity for the set of interphase and mitotic cells. In either case, the intensity level of CH_3_ signature is significantly higher than that of CH_2_ signature. It is 3.10-fold higher than that of CH_2_ for interphase cells (6.55 ± 1.62 and 2.11 ± 0.65, respectively), and 2.56-fold higher for mitotic cells (7.77 ± 2.26 and 3.04 ± 0.87, respectively). These results demonstrate that the biologically relevant complementarity between CH_2_ and CH_3_ vibrational signatures is obtained with high reproducibility. The CH_2_ stretching channel can reveal the ER organization in the cytoplasm. The CH_3_ one indicates the protein signal in the nucleus, highlighting nucleoli in particular.

### Investigation of all sub-phases of mitosis

**Figure 5:**
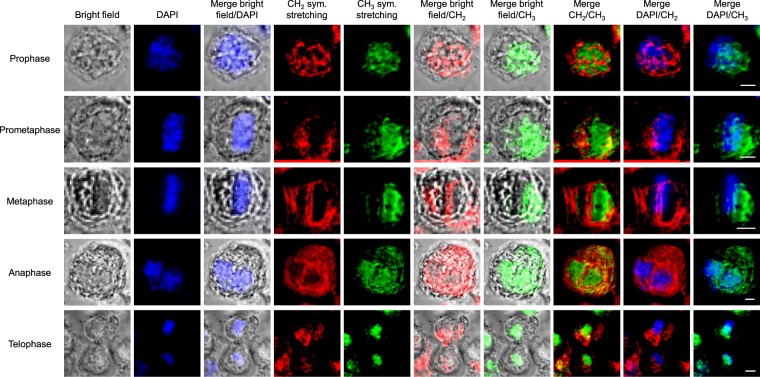
Analysis of DAPI-stained HEK293 cells in the different sub-phases of mitosis, including bright-field, fluorescence and MCARS (CH_2_, CH_3_) imaging. Scale bar, 5 µm.

The aforementioned methodology was applied to the analysis of DAPI-stained HEK293 cells in the different sub-phases of mitosis (see Fig. 5). To identify each sub-phase easily, cells were observed by fluorescence microscopy before operating MCARS microspectroscopy. It is obvious that, in all sub-phases of mitosis, CH_2_ and CH_3_ signatures do not overlap, in agreement with previous observations. Interestingly, DAPI and CH_3_ signals show a significant colocalization, since the lateral shift between both images in some cases (prophase, prometaphase, metaphase) is clearly due to the different physical principles of these modalities. In other words, there is an uncertainty in the choice of the focal plane for MCARS imaging with respect to that used for fluorescence microscopy. Consistently, CH_2_ vibrational signal is predominant in the cytoplasm and does not overlap with the fluorescence signal of DAPI, whatever the sub-phase. The CH_3_ vibrational signature allows following the movement of chromosomes during the different sub-phases of mitosis; e.g., the alignment of chromosomes in metaphase, their separation in anaphase, or the formation of chromosome clusters at cell opposite poles in telophase.

### Comparative analysis of fixed and living cells

**Figure 6:**
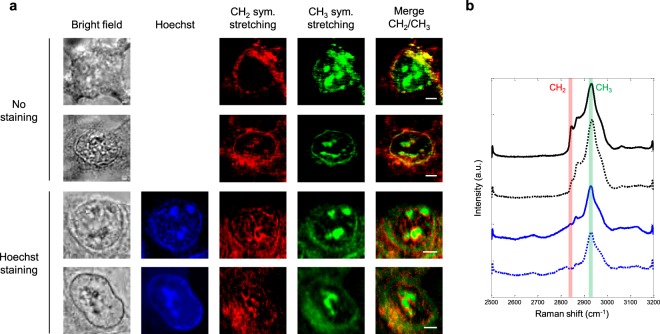
Analysis of interphase (G_1_/S), stained and unstained, living HEK293 cells. (**a**) Bright-field, fluorescence and MCARS (CH_2_, CH_3_, merge) images of selected cells. MCARS images were reconstructed at 2850 cm^−1^ (CH_2_ symmetric stretching) and 2930 cm^−1^ (CH_3_ symmetric stretching). Scale bar, 5 µm. (**b)** Standard deviation of the vibrationally resonant CARS signal between 2500 and 3200 cm^−1^, computed over the whole analysis window of each cell. Spectra are plotted vertically in the same order as corresponding cells. CH_2_ and CH_3_ channels are highlighted.

Hoechst 33342 was used for staining the nucleus of living HEK293 cells, since it allows efficient labelling without cell permeabilization. We first investigated interphase (G_1_/S), stained and unstained, living cells so as to assess the possible impact of Hoechst labelling on the vibrational signature in the CH stretching region. Actually, this fluorophore is expected to have specific Raman bands in the fingerprint region^32^, but its contribution in the high wavenumber region, even weak, must be considered^33^. Figure 6a shows bright-field, fluorescence and MCARS images obtained for two unstained (upper panel) and two stained (lower panel) cells. As previously, CH_2_ and CH_3_ signatures are complementary. Two particular features can be observed in the CH_3_ channel for both stained and unstained cells, namely the presence of bright intranuclear structures and the visualisation of nucleus border. Based on the images of Hoechst fluorescence, this corresponds to nucleoli and to heterochromatin contiguous to the nuclear envelop, respectively. Therefore, stained living cells can be accurately analysed by MCARS microspectroscopy in the 2500–3200 cm^−1^ range, since Hoechst labelling does not have any noteworthy effect on the vibrational signature. This assumption was corroborated by computing the standard deviation spectra of the vibrationally resonant CARS signal (Fig. 6b), which did not show substantial features due to Hoechst staining.

**Figure 7:**
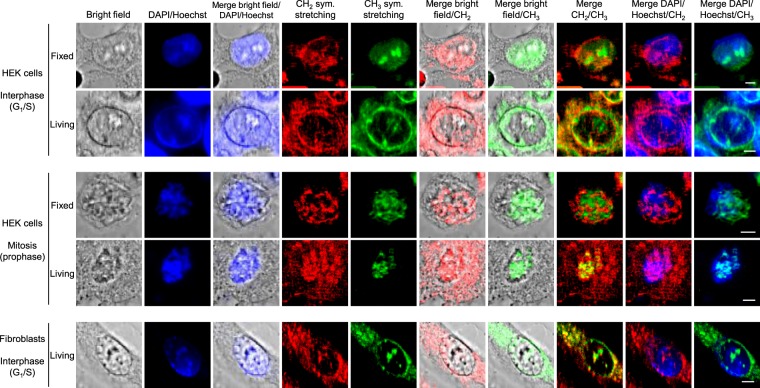
Analysis of interphase and mitotic, stained, fixed and living, HEK293 cells and fibroblasts, including bright-field, fluorescence and MCARS (CH_2_, CH_3_) imaging. Fixed and living HEK293 cells were stained with DAPI and Hoechst 33342, respectively. Living fibroblasts were stained with Hoechst 33342. Scale bar, 5 µm.

Then, we could compare fixed and living cells in the presence of DAPI and Hoechst staining, respectively. This study was realised for HEK293 cells and fibroblasts in interphase (G_1_/S) and mitosis (prophase), as presented in Fig. 7. In interphase, nucleoli are visible in the CH_3_ channel for both fixed and living cells, but the nucleus border is visible in the case of living cells only. This last observation was verified for a set of 10 fixed interphase cells (see Supplementary Fig. 1, where no nucleus border is visible) and 5 living interphase cells (see Figs 6a and 7, where the nucleus border is noticeable). Such phenomenon is therefore related to the cell fixation process and could be probed by means of MCARS microspectroscopy. In mitosis, only chromosomes are observed for both fixed and living cells, as expected.

## Discussion

In this work, we apply MCARS microspectroscopy in the CH stretching vibrational region (2500–3200 cm^−1^) to the analysis of a human cell line during the cell cycle. Generally, to observe the nucleus by vibrational technique through its DNA content, authors use the fingerprint region with Raman shifts at (1) 785 cm^−1^ corresponding to uracil/thymine ring breathing^2,6^, (2) 1095 cm^−1^ corresponding to phosphate symmetric stretching in the DNA backbone^8,19,34^, (3) 1487 cm^−1^ and 1578 cm^−1^ corresponding to adenine/guanine ring breathing, respectively^19,34^. Another strategy consists in focusing on proteins, which are the main component of heterochromatin (they represent two thirds of its mass). So, in view of the quantity of proteins present in heterochromatin, the analysis in the CH region is expected to provide stronger vibrational signatures compared to the fingerprint region, in favour of obtaining high contrast CARS images.

The difference in CH_3_ signature intensity between mitotic (prophase) and interphase (G_1_/S) cells (Fig. 4) may be due to the condensation state of chromatin: a strong condensation during mitosis (only heterochromatin) which requires a large amount of proteins such as histones, condensins and cohesins; and a lower condensation in interphase during which heterochromatin represents only a part of the whole chromatin in nucleus, mainly in nucleoli^35^. In this case, the rest of DNA is packaged in euchromatin, which is less condensed and includes a small amount of proteins, mostly histones. Besides, for interphase fixed (Figs 3 and 4) and living (Figs 6 and 7) cells, heterochromatin is depicted in the CH_3_ stretching channel as the nucleolar structures, corresponding to the bright spots observed in the nucleus with DAPI or Hoechst fluorescence, respectively. We cannot exclude that CH_3_ stretching at 2930 cm^−1^ corresponds mainly to proteins. It may also include a signal due to the presence of 5-metylcytosine, which is a hallmark of heterochromatin^36^.

Regarding the CH_2_ vibrational signature, it is primarily associated to lipids and therefore to plasma and organelle membranes. During the cell cycle, there is a remodelling and reorganization of the organelles in the cytoplasm. Among them, the ER corresponds to a large part of endogenous membranes. During interphase, the ER displays a typical reticular network of cisternae (sheets) and tubules, but it is predominantly organised as extended cisternae during mitosis^37^. This cisternal organisation of the ER is obvious for unstained prophase cells of Supplementary Fig. 2, indicating the effectiveness of MCARS microspectroscopy for probing the specific ER morphology during mitosis. Additionally, we could observe that the ER accumulates at the spindle poles and is excluded from the central area in prometaphase/metaphase^38^: this phenomenon is clearly visualised in the CH_2_ stretching channel for the prometaphase cell of Fig. 5.

We now discuss the impact of cell fixation on the visualisation of the nucleus border. For living cells only, the border of the nucleus is clearly visible through CH_3_ vibrational signature (Figs 6 and 7). This may be explained by the presence of heterochromatin, which binds indirectly to the nuclear envelope by means of proteins involved in tethering chromatin during interphase. Many nuclear envelope transmembrane proteins (NETs) binding chromatin have been identified, such as MAN1, LAP2β or emerin. They interact with chromatin proteins or methylated histones, and also with nuclear lamina^39,40^. Therefore, heterochromatin is retained at the inner nuclear membrane and allows to delimitate the nucleus in cell through its CH_3_ vibrational signature. When cells are fixed with PFA, the nuclear envelope is biochemically modified. The treatment may induce a loss of interaction between the NETs and the heterochromatin, which is no more connected to the inner nuclear membrane and may be released in the nucleoplasm. Only nucleoli, which contain a high quantity of heterochromatin, remain observable. These results are relevant in view of the numerous studies of cells fixed by PFA based on vibrational technologies.

For the simultaneous observation of chromosome movement during mitosis and of other structures in cell such as ER by epifluorescence, confocal, or two-photon microscopy, it is necessary to label DNA with specific dyes and cell compartment with specific antibodies. In both cases, cells must be permeabilized. Specific cell compartment labelling often requires the use of a primary antibody associated to a secondary one coupled to a fluorochrome. We show that MCARS microspectroscopy allows to directly visualize different cell components (chromosomes, ER) without labelling and cell permeabilization.

This work shows that high spectral resolution MCARS microspectroscopy in the high wavenumber region allows to visualise proliferation, the most common cellular process, with no prior labelling. By means of the vibrational signature of condensation proteins, structures like nucleoli, nuclear border and chromosomes can be identified. Furthermore, the structuration of the cytoplasm and especially the ER organisation can be effectively probed through the signature of lipids. It is also stressed that MCARS can reveal the impact of the fixative method on these structures. With this label-free imaging approach, it will be possible to visualize other cellular processes for which chromatin undergoes rearrangements.

## Methods

### Cell culture

Human embryonic kidney 293 (HEK293) cells were cultured in a DMEM medium (4.5 g/L of glucose, Gibco) supplemented with 10% (v/v) fetal calf serum (BioWest), 100 units/mL penicillin and 100 µg/mL streptomycin (Gibco) at 37 °C under humidified atmosphere and 5% CO_2_.

Fibroblast primary culture was seeded in a DMEM medium (4.5 g/L of glucose, Gibco) supplemented with amphotericin B at 1 µg/mL (Gibco), 100 units/mL penicillin and 100 µg/mL streptomycin and 10% (v/v) fetal calf serum at 37 °C under humidified atmosphere and 5% CO_2_.

HEK293 cells and fibroblasts were grown on uncoated glass slides to prevent generation of vibrational signal from the holder during the upcoming MCARS analysis. We used 12-well plates in which we introduced 18 mm diameter round glass coverslips. Cells were seeded at a density of 35.000 cells per well.

### Cell synchronization

To obtain populations of cells at G_1_/S boundary, we used a double thymidine block, since thymidine is inhibitor of DNA synthesis. After 48 h of culture in 12-well plate, cells were treated with thymidine at 2 mM during 18 h, released for 9 h by washing out the thymidine and then blocked again by addition in the culture medium of 2 mM of thymidine for 17 h^41^.

Concerning cell arrest in mitosis (prophase), after 48 h of culture, cells were treated with thymidine at 2 mM during 18 h. Then, cells were washed in DPBS (Dulbecco’s phosphate-buffered saline) and released for 3 h in complete medium allowing to enter S phase. Finally, cells were blocked with nocodazole at 100 ng/mL during 12 h^41^.

To analyse the different sub-phases of mitosis, cells were blocked in prophase as described above. After washing with DPBS to remove nocodazole, 1 mL of complete culture medium was added to the wells. Therefore, cells could advance synchronously in mitosis. Every 30 min, cells were washed and fixed.

### Cell fixation and staining

#### Unlabelled fixed cells

After synchronization, cells were washed three times with DPBS and fixed by PFA 4% (v/v) in DPBS during 10 min at room temperature. After three washings to eliminate PFA, the coverslip was sealed with nail polish on a microscopy glass slide.

#### Fixed cells with DAPI staining

After fixation and washing, the nuclei were labelled with 4′,6-diamidino-2-phenylindole (DAPI, Sigma-Aldrich) at 1 µg/mL for 5 min. Then the cells were washed, and the coverslip sealed as previously.

#### Living cells with Hoechst staining

Hoechst 33342 (Thermo Fisher Scientific) at 10 µg/mL was used during 15 min for staining living cells. Then the cells were washed, and the coverslip sealed as previously.

### Flow cytometry

Flow cytometry analysis was realized before (as a negative control) and after cell synchronization with 10^6^ cells. After centrifugation at 1500 RPM, the pellet was suspended in 300 µL of cold PBS and rapidly in 700 µL of cold ethanol (100%). The cell suspension was preserved at −20 °C overnight. Then cells were washed twice with DPBS, re-suspended with 1 mL of DPBS and treated with RNase A (Sigma-Aldrich) at 1 µg/mL for 20 min at room temperature. After that, cells were labelled with propidium iodide (0.5 mg/mL) and immediately analysed by means of FACSCalibur (Becton Dickinson) flow cytometer. The resulting cell distributions were computed by Modfit software in order to determine the proportion of cells in the different phases of the cell cycle.

### Fluorescence microscopy

Stained cells were observed with an epifluorescence microscope (Leica DMI4000B). Data were processed using MetaMorph software (Molecular Devices).

### MCARS microspectroscopy

The MCARS system is an adaptation, in forward configuration, of our epi-detected custom-built setup^42^. It is designed as follows. The pump source is a passively Q-switched microchip laser (Horus Laser, 1064 nm, 1 ns, 20 kHz, linearly polarized, <0.1 cm^−1^ linewidth). The laser beam is divided into two parts by using a half-wave plate and a Glan-Taylor polarizer. One part is injected into a photonic crystal fibre to generate a supercontinuum Stokes wave (600–1650 nm). This Stokes beam is collimated by means of a parabolic mirror and directed to a long-pass filter at 1050 nm (Thorlabs, FEL1050). The other part is used as the pump radiation of the CARS process after adjusting its power with a variable neutral density filter and its delay with a delay line. Then pump and Stokes beams are spatially combined through a notch dichroic beamsplitter (Semrock, NFD01-1064-25 × 36) and tightly focused onto the sample with a high numerical aperture microscope objective (Olympus, UPlanSApo 60x, N.A. = 1.2, water immersion). The position of the sample is controlled by means of a translation stage. The CARS signal generated by the sample is collected by a second microscope objective (Nikon, S Plan Fluor ELWD 60x, N.A. = 0.7), cleaned from any remaining pump signal by using a notch filter (Thorlabs, NF1064-44) and guided into a spectrometer (Horiba, LabRam HR Evolution, 600 gr/mm grating, Synapse CCD camera). In order to observe stained cells via the fluorescence of DAPI or Hoechst, the M-CARS system was complemented with a halogen light source, appropriate excitation and emission filters, and a dedicated CCD camera (Thorlabs, 1500M-GE).

The lateral, axial and spectral resolutions of the CARS microspectroscope are ~300 nm, 2 µm and 0.8 cm^−1^ respectively. During all experiments, the laser power of pump and Stokes radiations at the sample position was set at 55 mW and 9 mW, respectively. At this laser power (64 mW in total), no morphological change of cells was observed. This was confirmed by visualizing the sample with bright-field and/or fluorescence imaging.

After being selected by means of bright-field and/or fluorescence imaging, cells were analysed by MCARS microspectroscopy, using a lateral step of 300 nm for the cross-section mapping. Spectra were acquired from 2500 to 3200 cm^−1^ with 50 ms pixel dwell time and processed by using the MEM so as to extract the pure vibrationally resonant signal, which corresponds to the imaginary part of the third order nonlinear susceptibility (Im{*χ*^(3)^})^29^.

## Supplementary information

Supplementary Information

## Data Availability

The datasets generated during the current study are available from corresponding author P.L. (philippe.leproux@unilim.fr) on reasonable request.

## References

[1] van Manen H-J, Kraan YM, Roos D, Otto C (2005). Single-cell Raman and fluorescence microscopy reveal the association of lipid bodies with phagosomes in leukocytes. Proceedings of the National Academy of Sciences.

[2] Matthäus C, Chernenko T, Newmark JA, Warner CM, Diem M (2007). Label-Free Detection of Mitochondrial Distribution in Cells by Nonresonant Raman Microspectroscopy. Biophysical Journal.

[3] Scalfi-Happ C, Udart M, Hauser C, Rück A (2011). Investigation of lipid bodies in a colon carcinoma cell line by confocal Raman microscopy. Medical Laser Application.

[4] Abramczyk H (2015). The role of lipid droplets and adipocytes in cancer. Raman imaging of cell cultures: MCF10A, MCF7, and MDA-MB-231 compared to adipocytes in cancerous human breast tissue. The Analyst.

[5] Mariani MM (2010). Micro-Raman Detection of Nuclear Membrane Lipid Fluctuations in Senescent Epithelial Breast Cancer Cells. Analytical Chemistry.

[6] Kang J (2016). Investigating Effects of Proteasome Inhibitor on Multiple Myeloma Cells Using Confocal Raman Microscopy. Sensors.

[7] Zumbusch A, Holtom GR, Xie XS (1999). Three-Dimensional Vibrational Imaging by Coherent Anti-Stokes Raman Scattering. Physical Review Letters.

[8] Cheng J-X, Jia YK, Zheng G, Xie XS (2002). Laser-Scanning Coherent Anti-Stokes Raman Scattering Microscopy and Applications to Cell Biology. Biophysical Journal.

[9] Moura CC, Tare RS, Oreffo ROC, Mahajan S (2016). Raman spectroscopy and coherent anti-Stokes Raman scattering imaging: prospective tools for monitoring skeletal cells and skeletal regeneration. Journal of The Royal Society Interface.

[10] Weeks T, Schie I, den Hartigh LJ, Rutledge JC, Huser T (2011). Lipid-cell interactions in human monocytes investigated by doubly-resonant coherent anti-Stokes Raman scattering microscopy. Journal of Biomedical Optics.

[11] Nan X, Cheng J-X, Xie XS (2003). Vibrational imaging of lipid droplets in live fibroblast cells with coherent anti-Stokes Raman scattering microscopy. Journal of Lipid Research.

[12] Lee YJ (2014). Quantitative, Label-Free Characterization of Stem Cell Differentiation at the Single-Cell Level by Broadband Coherent Anti-Stokes Raman Scattering Microscopy. Tissue Engineering Part C: Methods.

[13] Le, T. T., Huff, T. B. & Cheng, J.-X. Coherent anti-Stokes Raman scattering imaging of lipids in cancer metastasis. *BMC Cancer* **9** (2009). 10.1186/1471-2407-9-42 PMC2640413 19183472

[14] Mitra, R., Chao, O., Urasaki, Y., Goodman, O. B. & Le, T. T. Detection of Lipid-Rich Prostate Circulating Tumour Cells with Coherent Anti-Stokes Raman Scattering Microscopy. *BMC Cancer* **12** (2012). 10.1186/1471-2407-12-540 PMC3519750 23171028

[15] Rodriguez LG, Lockett SJ, Holtom GR (2006). Coherent anti-stokes Raman scattering microscopy: A biological review. Cytometry Part A.

[16] Leproux P (2011). New opportunities offered by compact sub-nanosecond supercontinuum sources in ultra-broadband multiplex CARS microspectroscopy. Journal of Raman Spectroscopy.

[17] Okuno M, Kano H, Leproux P, Couderc V, Hamaguchi H (2008). Ultrabroadband multiplex CARS microspectroscopy and imaging using a subnanosecond supercontinuum light source in the deep near infrared. Optics Letters.

[18] Okuno M (2010). Quantitative CARS Molecular Fingerprinting of Single Living Cells with the Use of the Maximum Entropy Method. Angewandte Chemie International Edition.

[19] Parekh SH, Lee YJ, Aamer KA, Cicerone MT (2010). Label-Free Cellular Imaging by Broadband Coherent Anti-Stokes Raman Scattering Microscopy. Biophysical Journal.

[20] Camp CH (2014). High-speed coherent Raman fingerprint imaging of biological tissues. Nature Photonics.

[21] Czamara K (2019). Impact of cell cycle dynamics on pathology recognition: Raman imaging study. Journal of Biophotonics.

[22] Harper, J. V. & Brooks, G. The Mammalian Cell Cycle: An Overview. in *Cell Cycle**Control* **296**, 113–154 (Humana Press, 2004). 10.1385/1-59259-857-9:113 15576929

[23] Matthäus C, Boydston-White S, Miljković M, Romeo M, Diem M (2006). Raman and Infrared Microspectral Imaging of Mitotic Cells. Applied Spectroscopy.

[24] Pliss A, Kuzmin AN, Kachynski AV, Prasad PN (2010). Nonlinear Optical Imaging and Raman Microspectrometry of the Cell Nucleus throughout the Cell Cycle. Biophysical Journal.

[25] Karuna A (2019). Label-Free Volumetric Quantitative Imaging of the Human Somatic Cell Division by Hyperspectral Coherent Anti-Stokes Raman Scattering. Analytical Chemistry.

[26] Lu F-K (2015). Label-free DNA imaging *in vivo* with stimulated Raman scattering microscopy. Proceedings of the National Academy of Sciences.

[27] Yoneyama H (2018). Invited Article: CARS molecular fingerprinting using sub-100-ps microchip laser source with fiber amplifier. APL Photonics.

[28] Cadart C, Zlotek-Zlotkiewicz E, Le Berre M, Piel M, Matthews HK (2014). Exploring the Function of Cell Shape and Size during Mitosis. Developmental Cell.

[29] Vartiainen EM, Rinia HA, Müller M, Bonn M (2006). Direct extraction of Raman line-shapes from congested CARS spectra. Optics Express.

[30] Dou X (1998). Quantitative analysis of double-stranded DNA amplified by a polymerase chain reaction employing surface-enhanced Raman spectroscopy. Applied Optics.

[31] Krause M, Radt B, Rösch P, Popp J (2007). The investigation of single bacteria by means of fluorescence staining and Raman spectroscopy. Journal of Raman Spectroscopy.

[32] Uzunbajakava N, Otto C (2003). Combined Raman and continuous-wave-excited two-photon fluorescence cell imaging. Optics Letters.

[33] Pully VV, Lenferink A, Otto C (2009). Hybrid Rayleigh, Raman and two-photon excited fluorescence spectral confocal microscopy of living cells. Journal of Raman Spectroscopy.

[34] Matthews Q, Jirasek A, Lum J, Duan X, Brolo AG (2010). Variability in Raman Spectra of Single Human Tumor Cells Cultured *in vitro*: Correlation with Cell Cycle and Culture Confluency. Applied Spectroscopy.

[35] Lam YW (2005). The nucleolus. Journal of Cell Science.

[36] Lewis J, Bird A (1991). DNA methylation and chromatin structure. FEBS Letters.

[37] Lu L, Ladinsky MS, Kirchhausen T (2009). Cisternal Organization of the Endoplasmic Reticulum during Mitosis. Molecular Biology of the Cell.

[38] Schlaitz A-L (2014). Microtubules as key coordinators of nuclear envelope and endoplasmic reticulum dynamics during mitosis: Prospects & Overviews. BioEssays.

[39] Poleshko A, Katz RA (2014). Specifying peripheral heterochromatin during nuclear lamina reassembly. Nucleus.

[40] Czapiewski R, Robson MI, Schirmer EC (2016). Anchoring a Leviathan: How the Nuclear Membrane Tethers the Genome. Frontiers in Genetics.

[41] Whitfield ML (2000). Stem-Loop Binding Protein, the Protein That Binds the 3Ј End of Histone mRNA, Is Cell Cycle Regulated by Both Translational and Posttranslational Mechanisms. Molecular and Cellular Biology..

[42] Capitaine E (2018). Fast epi-detected broadband multiplex CARS and SHG imaging of mouse skull cells. Biomedical Optics Express.

