# Supplementary material for: Multiplex coherent anti-Stokes Raman scattering highlights state of chromatin condensation in CH region
Source: Sci Rep. 2019 Sep 25;9:13862. doi: 10.1038/s41598-019-50453-0 (PMC6761141; doi:10.1038/s41598-019-50453-0)

Supplementary Information

Multiplex coherent anti-Stokes Raman scattering highlights state of chromatin condensation in CH region

Tiffany Guerenne-Del Ben[1], Zakaniaina Rajaofara[2], Vincent Couderc[2], Vincent Sol[1], Hideaki Kano[3,4], Philippe Leproux[2,5,*], and Jean-Michel Petit[1,*]

[*] Correspondence to philippe.leproux@unilim.fr and jean-michel.petit@unilim.fr

[1] PEIRENE, EA 7500, University of Limoges, 123 avenue Albert Thomas, 87060 Limoges, France

[2] XLIM, UMR 7252, University of Limoges, 123 avenue Albert Thomas, 87060 Limoges, France

[3] Department of Applied Physics, Graduate School of Pure and Applied Sciences, University of Tsukuba, 1-1-1 Tennodai, Tsukuba, Ibaraki 305-8573, Japan

[4] Institute of Applied Physics, University of Tsukuba, 1-1-1 Tennodai, Tsukuba, Ibaraki 305-8573, Japan

[5] LEUKOS, 37 rue Henri Giffard, 87280 Limoges, France

Supplementary Fig. 1 Analysis of interphase, stained and unstained, fixed HEK293 cells. Bright-field, fluorescence and MCARS (CH_2, CH_3, merge) images of selected cells. MCARS images were reconstructed at 2850 cm^{-1} (CH_2 symmetric stretching) and 2930 cm^{-1} (CH_3 symmetric stretching). Scale bar, 5 µm.

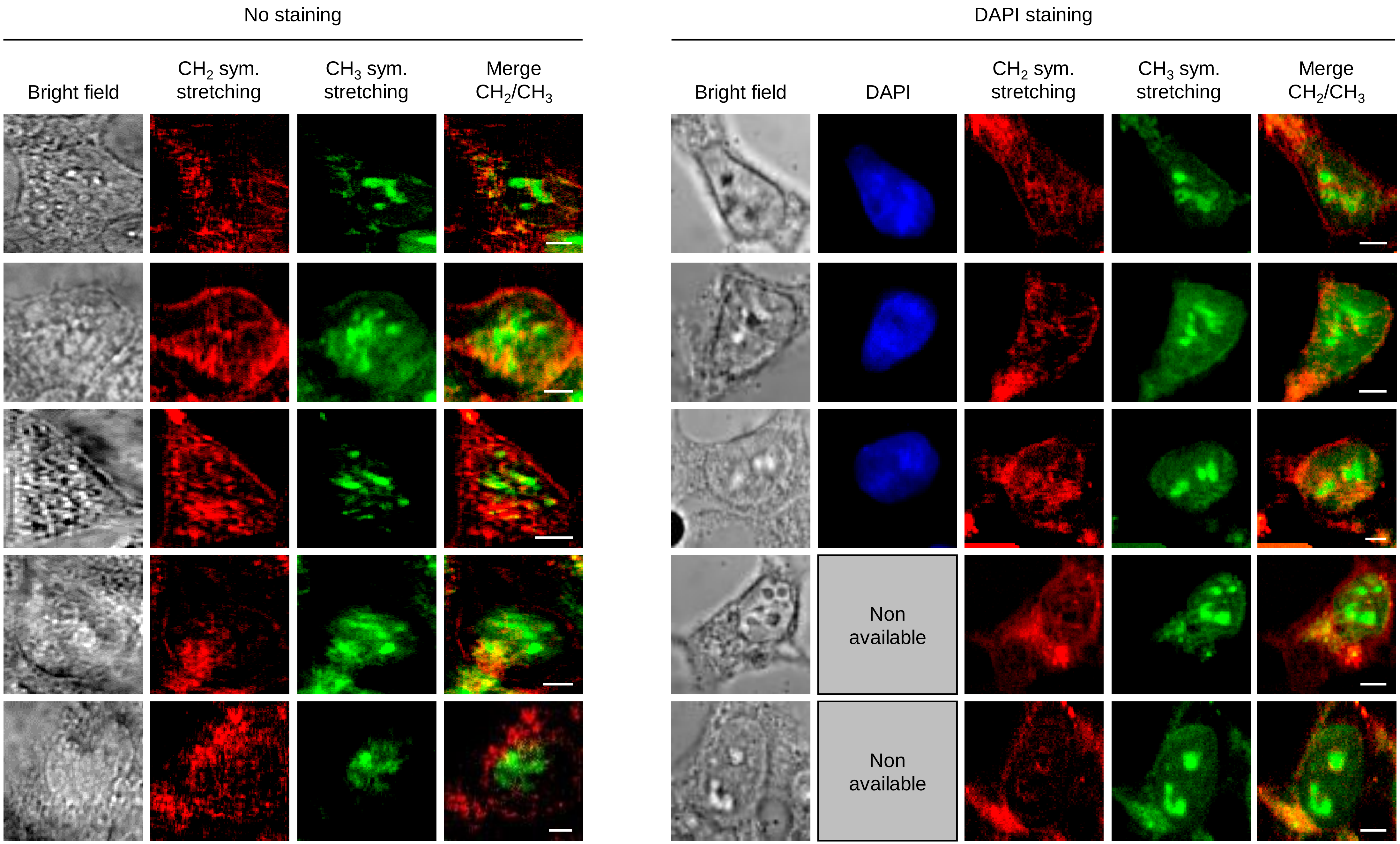

Supplementary Fig. 2 Analysis of mitotic, stained and unstained, fixed HEK293 cells. Bright-field, fluorescence and MCARS (CH_2, CH_3, merge) images of selected cells. MCARS images were reconstructed at 2850 cm^{-1} (CH_2 symmetric stretching) and 2930 cm^{-1} (CH_3 symmetric stretching). Scale bar, 5 μm.

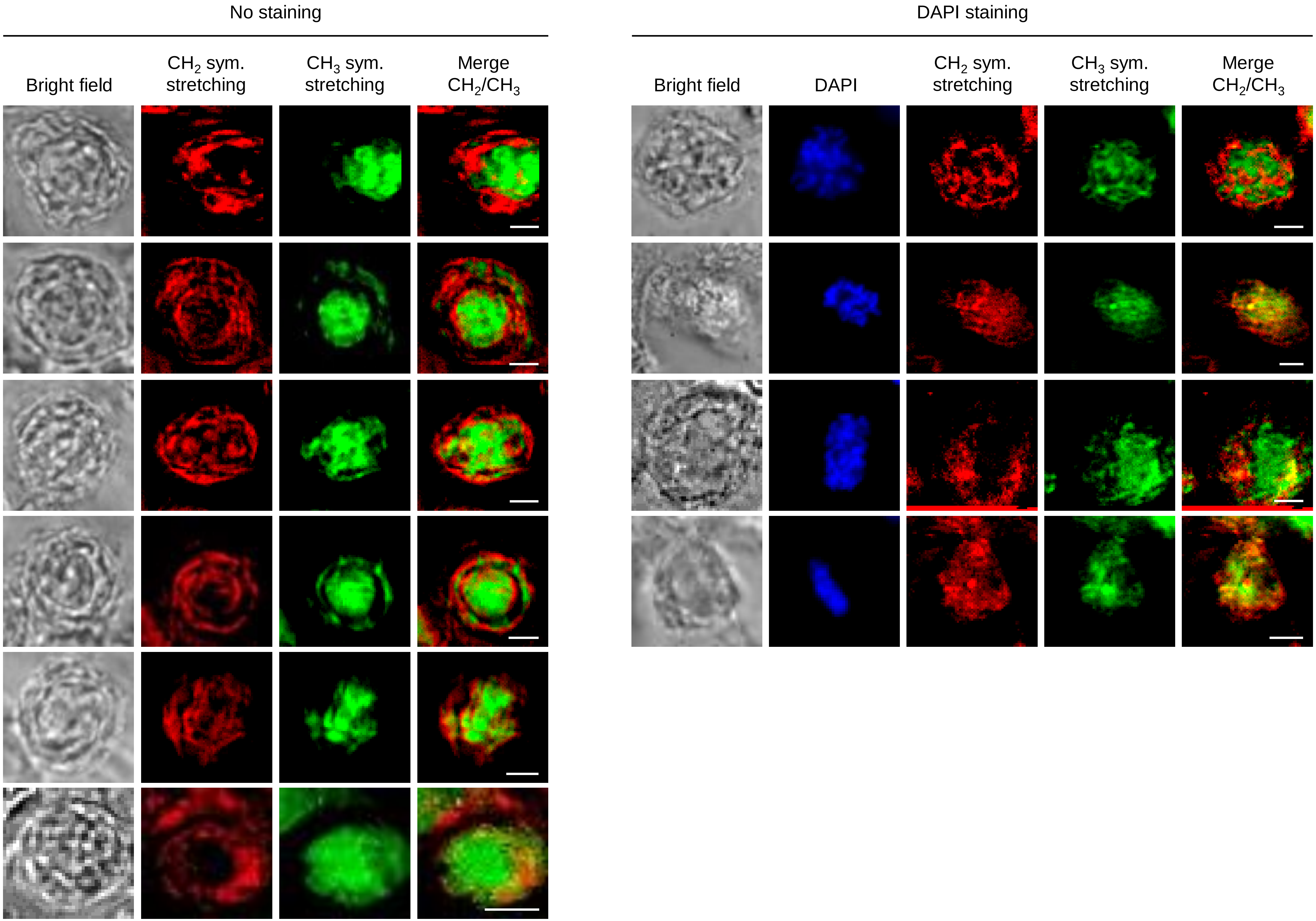

Supplement: Supplementary file 1 — Supplementary Information [file 41598_2019_50453_MOESM1_ESM.pdf]
